# Women’s health during the COVID-19 pandemic

**DOI:** 10.4069/kjwhn.2020.06.10

**Published:** 2020-06-17

**Authors:** Dong-Hyun Kim

**Affiliations:** Department of Social and Preventive Medicine, Hallym University College of Medicine, Chuncheon, Korea

**Keywords:** COVID-19, Health equity, Pandemics, Public health, Women

## Health effects of the COVID-19 pandemic by sex

As of June 9, 2020, the total worldwide number of confirmed cases of coronavirus disease 2019 (COVID-19) has passed 7 million, with over 400,000 deaths. Since the daily number of new confirmed cases is still increasing, it would be unrealistic to say that the pandemic has passed its peak. The public health crisis due to COVID-19 is affecting every citizen in most countries throughout the world, regardless of age, sex, and race. Abundant research and media coverage have addressed the differential effects of this pandemic according to age and race, including the fact that elderly individuals and racial minorities face higher risks of dying from COVID-19. However, fewer studies have analyzed the roles of sex and gender in the COVID-19 pandemic, and these parameters are not very clearly visible on some dashboards, despite consistent evidence regarding the impacts of sex (biological factors) and gender (social factors) on health outcomes [1].

Although men appear to be slightly more likely than women to contract COVID-19 globally [1], the contrary is observed in South Korea. According to the daily official reports of the Korea Centers for Disease Control and Prevention, there have been more cases in women (58%) than in men (42%) among the total of 11,852 confirmed cases in Korea as of June 9, 2020 [2]. The ratio of women to men increases with age, and is higher than 1 in all groups aged 20 or over. Of particular note, women in the oldest old category (80 years of age or older) appear to be diagnosed with COVID-19 2.2 times more frequently than men (data not shown). This discrepancy reflects the presence of more women residents in hard-hit facilities, such as long-term care facilities and care homes for older adults, and the overrepresentation of women in densely populated workplaces and in frontline healthcare settings among young and middle-aged adults.

It has been repeatedly reported that once infected, men are more likely to die than women. However, this may not be true depending on the indicator used when making this comparison. Several indicators can be used to assess and compare the mortality burden of COVID-19, such as the case-fatality rate (CFR), population mortality per million, and excess mortality. It has clearly been established that the CFR, which is the total number of deaths divided by the total number of confirmed cases, is higher in men than in women. In South Korea, there have been 274 deaths due to COVID-19 (146 men vs. 128 women), and the CFR is 2.93% in men and 1.86% in women [2]. The overall rate ratio for the sex-specific CFR is 1.58. The mortality rate per million, which measures the probability of any individual in the population dying from COVID-19, has a smaller rate ratio of 1.13 (5.62 per million for men and 4.98 per million for women). However, the estimates for those two indicators may be biased due to the availability of testing capacity, differences in public health strategies for testing, and the accuracy of death reports [3]. To overcome these limitations, Krieger et al. [4] compared the overall excess of deaths compared to the same time period in previous years between women and men, using Massachusetts mortality data. They observed a sharp rise in total excess mortality during the first two weeks of April 2020, but no material differences between men and women, as the age-standardized rate ratio for 2020 versus 2015–2019 was 1.48 for women and 1.55 for men. Considering the minimal difference in excess mortality by sex and the higher prevalence of comorbid conditions (e.g., hypertension and diabetes mellitus) and risk behaviors (e.g., smoking and less handwashing) among men, the direct and indirect effects of COVID-19 on health may be stronger for women than men.

## Gendered impact in public health preparedness and response to the COVID-19 crisis

Incorporating gender into the assessment of public health preparedness and institutional responses to pandemic crises such as COVID-19 is important for increasing the effectiveness of interventions and for promoting health equity among men and women, since the impact of COVID-19 is not only determined by biological differences. Kim et al. [5] analyzed gendered aspects of the COVID-19 outbreak and response in terms of quarantine and mitigation policies in Korea. They pointed out that the “declaration of war” against COVID-19 and the society-wide campaign “thanks for” led to a masculine atmosphere. The dedication of frontline healthcare workers was highly appreciated, as shown by references to their heroic activities, but social issues such as overwork, burnout, and the work environment were neglected. Notably, the voices of women healthcare workers, mostly nurses, were not heard and rarely covered in major media. As of April 5, among a total of 241 healthcare workers infected by COVID-19 [6], 190 (78.8%) were nurses, most of whom were women. Furthermore, women comprise the majority of care workers at high-risk facilities, such as long-term care facilities and nursing homes, where personal protective equipment, including masks, were not provided in the initial phase of this outbreak. This may have contributed, at least in part, to the repeated COVID-19 clusters at these facilities.

The impact of social distancing measures adopted by every country as a mitigation policy has revealed the reality of social inequality toward women. Temporary workers, a socially vulnerable group in this crisis, have been more likely to lose their jobs and tend to earn less than full-time regular workers. In South Korea, a higher proportion of women are temporary workers (45.0%) than men (29.4%) [7]. Economic hardship caused by this public health crisis has differential effects according to gender through already built-in social mechanisms of inequality. With the closure of schools and workplaces, women face a larger burden of unpaid work and housework. Single parents, who are mostly women, will face even greater difficulties in coping with a more stressful working environment and a heightened childcare burden due to social distancing measures. These factors may all aggravate women’s health both immediately and in the long term, unless specific actions are taken to alleviate and interrupt this vicious circle.

## Need for public health policies with a gender perspective during and after the pandemic

Public health crises like COVID-19 raise several issues for every society, including medical, public health, and social issues. Gender is one of the most important lenses to analyze and tackle these issues, one by one, efficiently and equitably. Policymakers and public health experts in charge of preparing and responding to the COVID-19 pandemic need to have a gender perspective. Gender-based data analyses are urgently needed to address which groups of women—and men—are the most vulnerable during this situation and to develop gender-sensitive and gender-balanced public health policies.

## References

[1] Global Health 5050 Sex, gender and COVID-19: overview and resources [Internet]. https://globalhealth5050.org/covid19/.

[2] Ministry of Health and Welfare, Korea (2020). Updates on COVID-19 in Republic of Korea (as of 2020 Jun 9) [Internet]. http://ncov.mohw.go.kr/bdBoardList_Real.do?brdId=1&brdGubun=11&ncvContSeq=&contSeq=&board_id=&gubun=.

[3] Kim DH, Choe YJ, Jeong JY (2020). Understanding and interpretation of case fatality rate of coronavirus disease 2019. J Korean Med Sci.

[4] Krieger N, Chen JT, Waterman PD (2020). Excess mortality in men and women in Massachusetts during the COVID-19 pandemic. Lancet.

[5] Kim S, Kim JH, Park Y, Kim S, Kim CY Gender analysis of COVID-19 outbreak in South Korea: a common challenge and call for action [published online ahead of print, 2020 May 22]. Health Educ Behav.

[6] Ministry of Health and Welfare, Korea (2020). Regular briefing of Central Disaster and Safety Countermeasure Headquarters on COVID-19 (as of 2020 Apr 7) [Internet]. http://ncov.mohw.go.kr/en/tcmBoardView.do?brdId=12&brdGubun=125&dataGubun=&ncvContSeq=353968&contSeq=353968&board_id=1365&gubun=.

[7] Statistics Korea (2019). Economically Activity Population Additional Survey: Paid workers by status of employment (by sex/age·educational attainment·marital status) [Internet]. https://gsis.kwdi.re.kr/statHtml/statHtml.do?orgId=338&tblId=DT_1XD9002&conn_path=I2&language=en.

